# Supplementary material for: Immune Profile Determines Response to Vaccination against COVID-19 in Kidney Transplant Recipients
Source: Vaccines (Basel). 2023 Oct 11;11(10):1583. doi: 10.3390/vaccines11101583 (PMC10611345; doi:10.3390/vaccines11101583)
Supplement: Supplementary file 1 [file vaccines-11-01583-s001.zip › vaccines-2607971-supplementary.pdf]

Supplement Figures

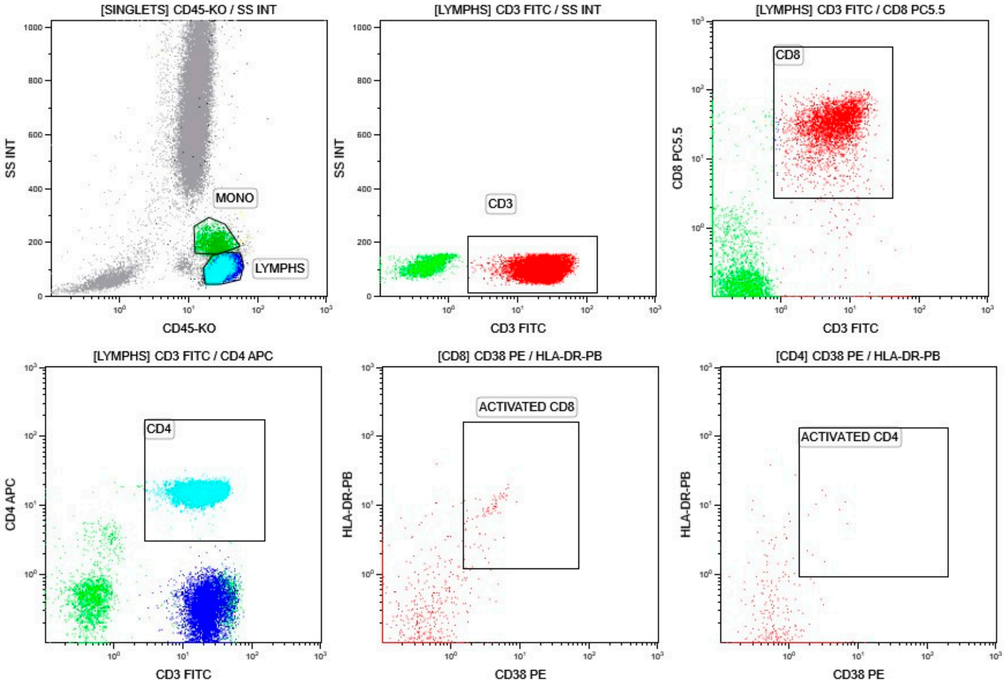

Supplement Figure S1. Gating strategy for CD3, CD4, CD8 and Activated CD4, Activated CD8 respectively

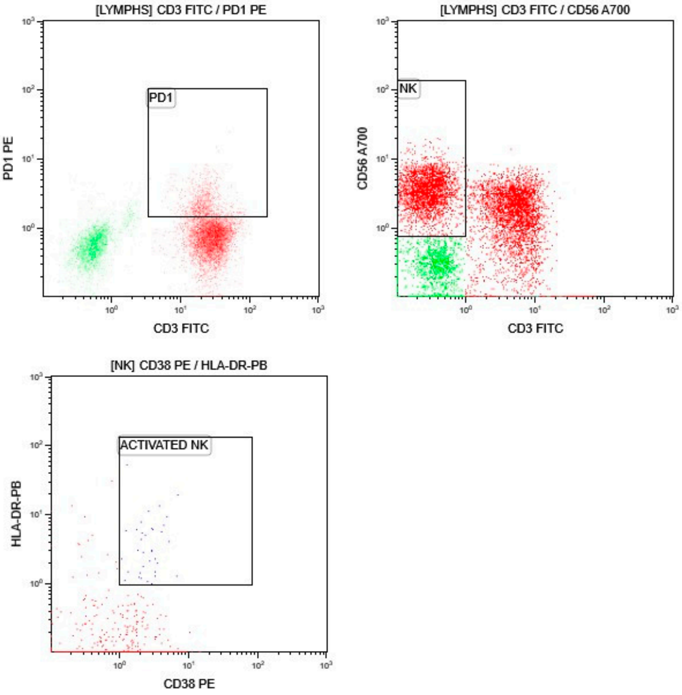

Supplement Figure S2. Gating strategy for CD3-CD56+ (Natural Killer - NK) cells and Activated NK cells

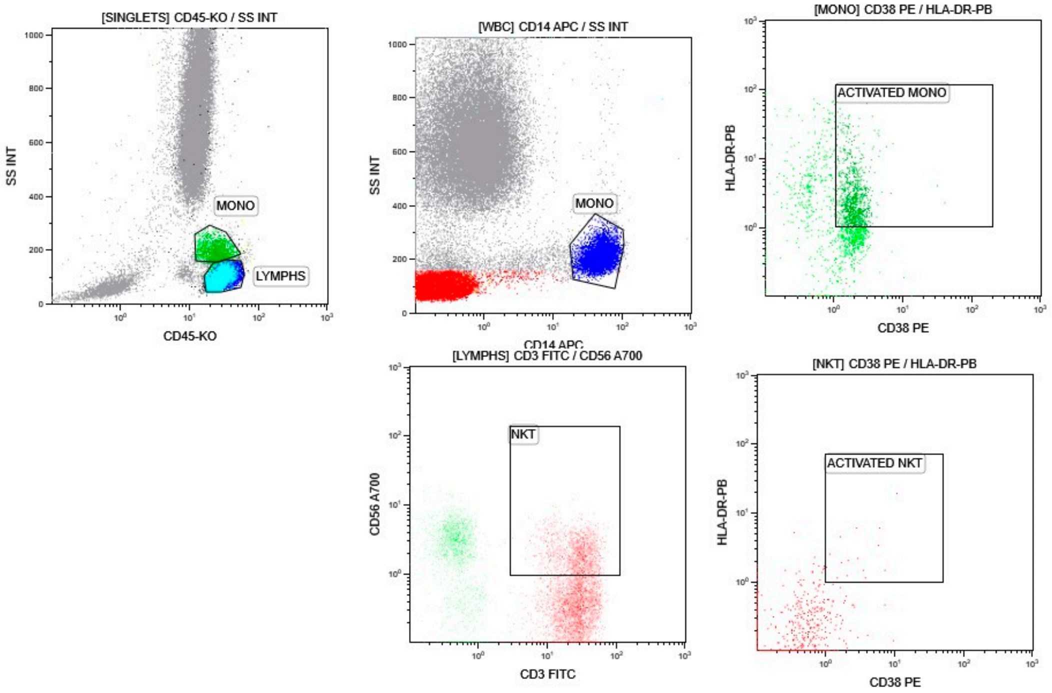

Supplement Figure S3. Gating strategy for Monocytes and Activated Monocytes, CD3+CD56+ (NKT cells) and Activated NKT cells

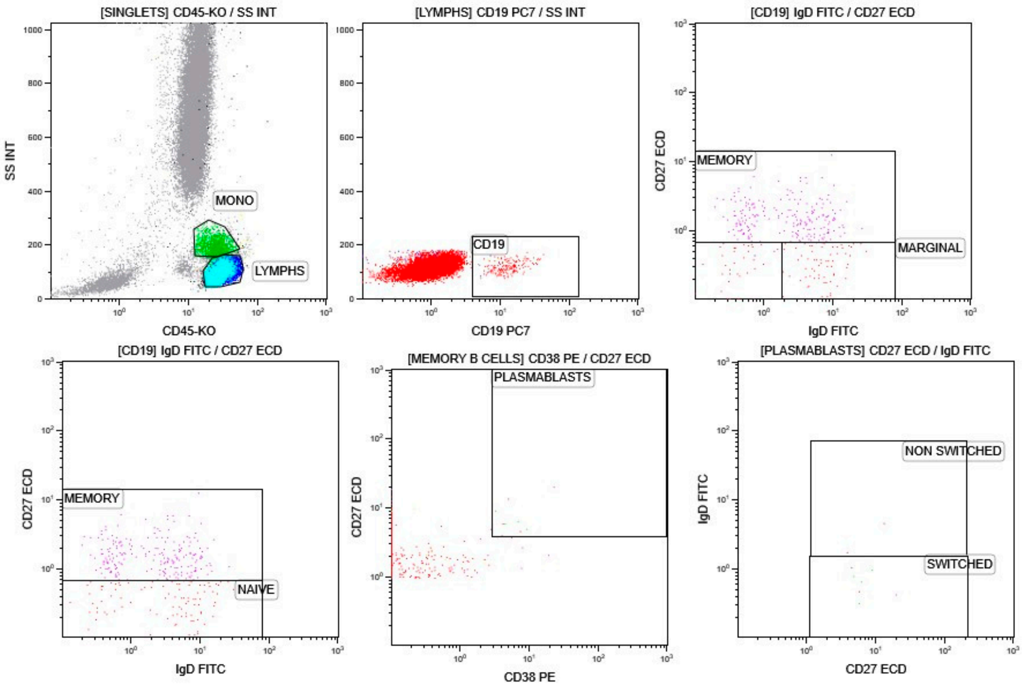

Supplement Figure S4. Gating strategy for CD19+ cells, CD19+CD27- (naïve), CD19+CD27+ (memory), CD19+ CD27-IgD+ (marginal), CD19+CD27+CD38+(plasmablasts), CD19+CD27+IgD+ (non switched) and CD19+CD27+IgD-(switched) cells.
